# Moroccan *Citrus clementina* Peels: Optimization of Pectin Extraction and Determination of Chemical and Functional Properties

**DOI:** 10.3390/plants12193417

**Published:** 2023-09-28

**Authors:** Hanane Azzouzi, Loubna Elhajji, Mouad Achchoub, Souad Salmaoui, Abdelillah Ammadi, Hasnaa Harrak, Rachid Touzani, Younes Noutfia, Kaoutar Elfazazi

**Affiliations:** 1 National Institute of Agricultural Research (INRA), Avenue Ennasr, BP 415 Rabat Principal, Rabat 10090, Morocco; hananeazzouzi94@gmail.com (H.A.); achchoubmouad1992@gmail.com (M.A.); ammadiabdelillah@gmail.com (A.A.); hasnaa.harrak@inra.ma (H.H.); younes.noutfia@inra.ma (Y.N.); 2 Laboratory of Environmental Engineering, Ecology and Agro-Industry, Faculty of Science and Technology, Sultan Moulay Slimane University, Beni Mellal 23000, Morocco; elhajjiloubna.1989@gmail.com (L.E.); souadsalmaoui@yahoo.fr (S.S.); 3 Laboratory of Environment and Applied Chemistry (LCAE), Faculty of Sciences, Mohammed First University, Oujda 60000, Morocco; r.touzani@ump.ac.ma

**Keywords:** citrus by-products, pectin extraction, optimization, characterization, response surface methodology, functional properties

## Abstract

Citrus peels are considered a rich source of valuable biomolecules. Pectin is a polymer of polysaccharide acid and is composed of galacturonic acid monosaccharides. In this study, response surface methodology was used to optimize pectin extraction from *Citrus × clementina* Hort. ex Tan. (Rutaceae) peels using citric acid as an extraction solvent. The effect of the parameters conditioning the extraction process and pectin yield (pH level, temperature, extraction time, solid/liquid ratio, and raw material particle size) was investigated using a Box–Behnken design. The quality of the extracted pectin was assessed both chemically (moisture, ash, protein, and carbohydrate content) and functionally (gelling power and emulsifying activity). According to the screening experiment, the pH level, temperature, and particle size were the main factors influencing the pectin yield. The adjusted mathematical model enabled us to plot response surfaces in order to determine the optimal extraction conditions. The highest production yield of pectin (26.6%) was obtained at the optimal conditions of pH = 1.5, temperature = 100 °C, and particle size = 0.1 mm for an extraction time of 30 min. Compared to the predicted value of 26.6%, the experimental extraction yield of *C. clementina* was about 21.4% of pectin. Concerning the functional properties, the extracted pectin had a high gelling power of 164 ° SAG and an emulsifying activity of 38.5%.

## 1. Introduction

Many countries around the world depend on the socioeconomic contribution of the citrus industry. In Morocco, approximately 2.62 million tons of citrus are produced, of which 715,000 tons are destined for export, representing a value of more than USD 432 million. Among these citrus fruits, clementines represent about 25% of the total planted area [1].

Citrus fruit production is intended for either direct consumption (fresh) or processing (drinks, jams, etc.) [2]. Citrus juice manufacturing generates a significant amount of waste and by-products. These by-products are underexploited in the agri-food sector and generally consumed as animal feed or compost fertilizers. However, these by-products are valuable sources of natural products, such as essential oils, phenolic compounds, flavonoids, and polysaccharides like cellulose, or more specifically, pectin [3].

Pectins are a class of complex polysaccharides that are found in plant cell walls. They are classified as food additives (registration number E440). The pectin chains are largely made up of α-(1→4)-linked D-galacturonic acid units, connected by glycosidic linkages and a small amount of branched L-rhamnose [4].

In the food industry, pectins are widely applied as thickeners, gelling agents, and emulsifiers in jams, jellies, fruit juices, desserts, and dairy products [5]. They are also used as reinforcement in biomaterials [6] and as an encapsulation agent for active substances in various fields (pharmaceuticals, agri-foods, cosmetics, etc.) thanks to their ability to envelop compounds of interest, such as flavors and vitamins [7]. The global demand for pectin exceeds 30,000 tons annually and is growing at about 4–5% per year [8].

Commercial pectin may be obtained by extractions from various plant sources, mainly from apple pomace and citrus peels [9]. The extraction process includes hydrolyzing protopectin using acids, such as sulfuric, phosphoric, nitric, hydrochloric, or citric acid, at high temperatures [10]. Many reports have demonstrated that the factors affecting pectin extraction are pH level, temperature, extraction time, particle size, type of acid, and solvent-to-sample ratio (SSR) [2,11]. Furthermore, researchers have optimized the yield of these extraction conditions using response surface methodology (RSM) [2,11]. RSM can be an effective tool for optimizing experimental conditions for a specific response while using a minimum number of experiments [12].

RSM helps reduce experimental trials and perform multiple-factor analysis to optimize conditions for pectin extraction [13]. However, to our knowledge, no previous data have been published on extracting pectin from Moroccan *Citrus clementina* Hort. ex Tan. (Rutaceae) peel. Therefore, the current study aims to (i) identify the optimal acidic conditions for the extraction of pectin from clementine by-products to obtain the maximum yield using a three-variable Box–Behnken response surface design and (ii) investigate the chemical and functional properties of the extracted pectin.

## 2. Results and Discussion

### 2.1. Experimental Design

Substituting the variables coded as −1, +1, and 0 for real values, the experimental conditions, and the corresponding experimental responses (pectin yield) were obtained and are presented in Table 1. Fourteen experiments with three independent variables (pH level, temperature, and particle size) were performed to evaluate the corresponding pectin yield.

A similar result reported by Aina [14] shows that the maximum yield from *Citrus sinensis* was found to be 29.41% at pH 3.2 and a temperature of 70 °C. The optimized extraction from *C. Limon* resulted in a maximum yield of 36.71% at pH 3.2 and a temperature of 60 °C [15].

Thus, a pH of 3.2 appears to be optimal for the extraction of pectin from all the citrus peels studied. The optimal temperature for pectin extraction was observed to be 70 °C for *C. sinensis* and *C. limetta*, but not for *C. Limon*, in which case a lower temperature of 60 °C was preferred [15].

### 2.2. Estimated Model

The observed responses were used to compute the model coefficients using the least square method. This allowed us to establish the following estimated model:

Yield = 15.18 − 2.43 × X_1_−7.48 × X_2_ + 1.55 × X_3_ (1)

### 2.3. Statistical Analysis and Validation of the Model

The statistical ‘model fit summary’ confirms the suitability of the linear model compared with the quadratic or 2FI model (Table 2). Also, the predicted R² of 0.8556 and the insignificant lack of fit imply that the linear model was the best model to represent the parametric effects on the yield (Table 3).

The results illustrated in Table 4 indicate that the model F-value of 46.45 implies that the model is significant. Model terms are significant when the *p*-value is less than 0.0500. In this case, X1, X2, and X3 are significant model terms, and values greater than 0.1000 reveal that the model terms are not significant. The lack-of-fit F-value of 818.41 implies that the lack of fit is significant.

The predicted R^2^ of 0.8556 is in reasonable agreement with the adjusted R² of 0.9130, i.e., the difference is less than 0.2. A ratio greater than 4 is desirable. The ratio of 19.301 indicates an adequate signal (Table 5). Overall, this model can be used to navigate the design space.

The coefficient estimate represented in the Table 6 reveals the expected change in a factor’s value per unit of response when the other factors are maintained as constant. The average response of all the runs is the intercept in an orthogonal design. Based on the factor settings, the coefficients are changed to approximate the average. The variance inflation factor (VIF) is one if the factors are orthogonal. Multi-linearity is indicated by VIFs greater than 1, and we have a stronger correlation with a higher VIF. VIFs less than 10 are generally acceptable.

### 2.4. Interpretation of the Response Surface Model

**Figure 1:**
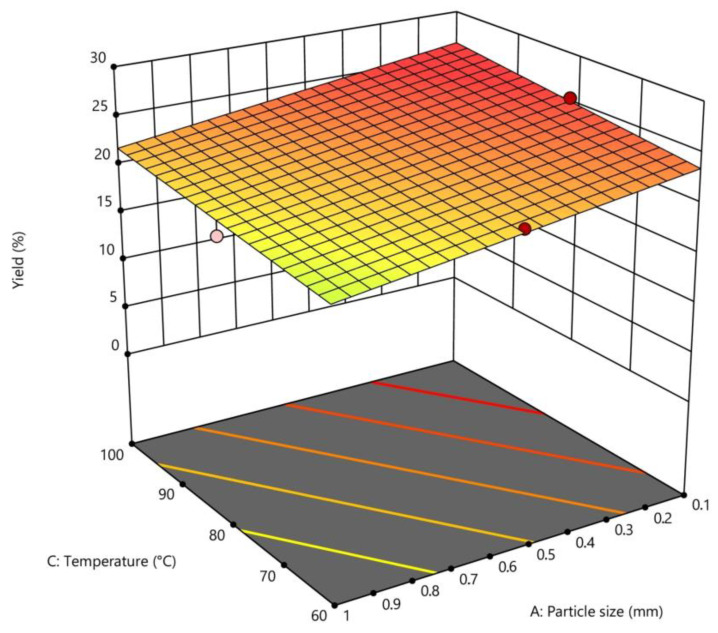
Three-dimensional response surface for the effect of temperature extraction and particle size at constant pH (1.5) on pectin yield extracted from dried *Citrus clementina* peels.

**Figure 2:**
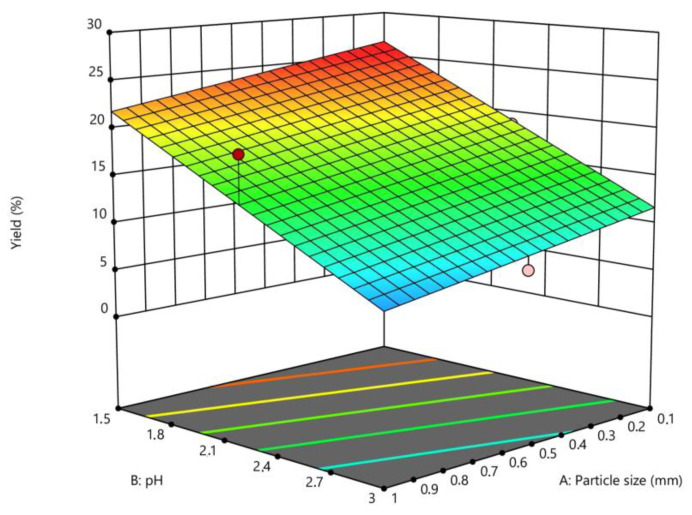
Three-dimensional response surface for the effect of pH extraction and particle size at constant temperature (100 °C) on pectin yield extracted from dried *Citrus clementina* peels.

The response surface model graphically illustrates the relationship between the response and the experimental variables by establishing three-dimensional plots [2]. The graphics presenting the relationship between the response (extraction yield) and the experimental variables (pH level, temperature, particle size) are shown in Figure 1 and Figure 2. Each of the two horizontal axes represents one of the three independent variables, while the vertical axes reflect the pectin extraction yield (%). By fixing a factor either in its lower or upper level, or at its average value, a graphical representation of the response as a function of the other two factors can be displayed.

The pectin yield variation as a function of pH variation (1.5 to 3) indicates that the pectin yield increased when the pH level decreased. This could be attributed to the fact that, at low pH, citric acid can hydrolyze insoluble pectin and transform it into its soluble form, thus increasing the yield of pectin extraction [16]. Furthermore, low pH may decrease the molecular weight of pectin, and thus increase its release from the plant tissue without degradation [17,18]. Similar results were published on apple pomace, sugar beet pulp, mango peel, and pomegranate peel by Canteri-Schemin [19], Yapo [20], Prakash Maran [5], and Moorthy [17], respectively.

Temperature is considered to be one of the most crucial parameters affecting the pectin extraction yield. The effect of temperature is shown in Figure 1. The results show that when the pH was fixed at 1.5, the extraction yield increased relative to the increasing temperature.

According to Yang [21], the extraction yield of pectin increases when temperature increases due to the solvent’s enhanced solubility and diffusivity in the plant tissue. These results are similar to those observed by Pagan [22] and Raji [11], who reported a significant improvement in the yield of pectin extraction from peach pomace and melon rind, respectively, with increasing temperature [18].

The results shown in Figure 2 reveal that particle size has a substantial impact on pectin yield. At a fixed temperature of 100 °C, the production of pectin was greater for the 0.1 mm sized raw material (26.64%). This could be because protopectin is more commonly available in small particles than in large ones. In other words, when the particle size is reduced, the surface area increases, which increases the exposure to the extracting agent [19].

### 2.5. Determination of Optimum Conditions

To select the optimal conditions, the particle size value was fixed at 0.1 mm, and pH was plotted versus temperature. The conditions that obtained the highest yield (Y1 = 26.64%) were a temperature of 100 °C, a pH of 1.5, a grain size of 0.1 mm, a solid/liquid ratio of 1:50 (*m*/*v*), and an extraction time of 30 min.

The validation experiments that were performed under the selected conditions obtained an experimental yield of pectin (21.36%) that was lower than that calculated mathematically by the optimization model (26.64%). This difference of about 5% between the theoretical and experimental values may be due to the difficulty of controlling the stability and precision of the experimental conditions.

Many authors have reported that pH, temperature, extraction time, particle size, agitation, and solid-to-liquid ratio have effects on the yield and the quality of pectin [11,14,16,22]. Pectin has been extracted from many plants, including lemon, orange, peach pomace, and sugar beet pulp, and characterized. Pagan (1999) [22] extracted pectin from fresh peach pomace under different experimental conditions and found that the highest yields were obtained at the highest temperatures and at the lowest assayed pH level. El-Nawawi (1987) [16], who studied the effect of experimental conditions on the yield of pectin extracted from Egyptian orange, found an optimum yield at 90 °C, a pH of 1.7, and 2 h of extraction.

Other factors influence the yield of pectin, including the stage of maturity and the drying process. The pectin yield tends to increase as the fruit matures, which aligns with earlier findings indicating that the galacturonic acid levels in fruit peel increase alongside the overall insoluble carbohydrate content as the fruit matures [23]. While limited research is available regarding the effects of drying temperature and methods on pectin, one study noted an increase in pectin yield when the drying temperature was elevated from 40 to 70 °C. However, other studies found no alteration in the structural and functional properties of pectin as a result of different drying methods [24].

### 2.6. Chemical Characteristics

**Figure 3:**
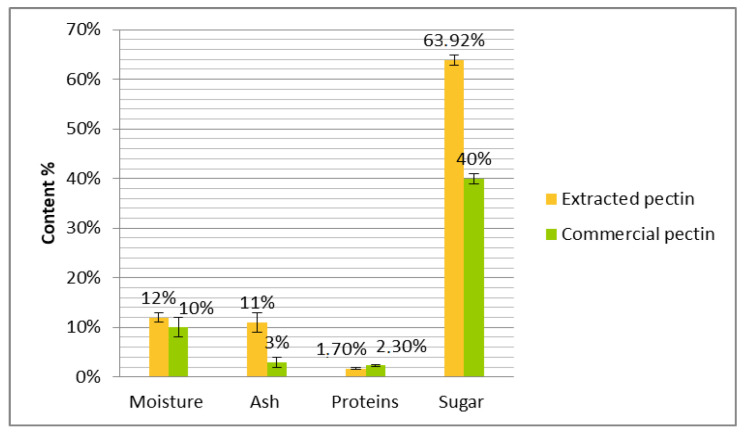
Chemical characterization of pectin extracted from clementine compared to commercial pectin.

The comparison between extracted and commercial pectin based on moisture, ash, protein, and sugar contents is reported in Figure 3.

The moisture content of the extracted pectin was about 12%. This value is comparable to that reported by Baississe (2009) [25], who used aluminum chloride and aluminum sulfate to extract pectin from oranges and found moisture values of 8 and 13%. These values are still acceptable for good storage stability [25]. When compared to commercial pectin, the moisture content of extracted pectin is slightly higher, by 2%. This difference may be attributed mainly to extraction conditions, species of used citrus, etc.

The ash content of the pectin obtained from clementines was 10.6%. This value is significantly higher than the commercial pectin (3%), which can be explained by the fact that, in our extraction, we used citric acid as the extraction solvent; commercial pectin uses powerful acidic agents to extract and purify pectin. This result is similar to that obtained by Baississe [25], who showed that the ash content of pectin precipitated by mineral salts, especially aluminum sulfate and aluminum chloride, varied between 8 and 25%. Extraction conditions can also affect the precipitation of impurities with pectin and impact ash content. Thus, Yapo et al. [20] concluded that pectin extracted at pH 1.5 is purer than that obtained by solubilization at pH 2.0 from beet pulp. Khotimchenko et al. [26] showed that the sorption activity of pectin towards heavy metals is closely related to pH, where it varies in the range of 4–8.

The maximum protein content in pectin is 2.5%, according to LEU and FAO/WHO, which are cited by Herbstreith and Fox [27]. Regarding the recorded value of 1.7% obtained in this study, this indicates that the pectin extracted from clementines is still within the permitted range of 2.5%. The pectin extracted from apple pomace by Massiot and Renard [28] showed a protein content ranging from 2.1 to 7.5%. Yapo et al. [20] obtained a protein content of 3.7% in pectin extracted from beet pulp by hydrochloric acid.

Moreover, the percentage of total carbohydrate in the extracted pectin was 63.9%, which is higher than that obtained for commercial pectin (40%). These results are comparable with those described by Baississe [25], who reported values of 70.8% and 74.1% for total carbohydrate of pectin extracted by aluminum chloride and aluminum sulfate, respectively. The study of Lekbir [29] showed lower values than those of our study, with a total sugar content that varied between 24.2 and 18.9% for orange pectin precipitated with aluminum chloride and aluminum sulfate, respectively. Several studies have shown that parietal polysaccharides, especially pectin, are subject to qualitative and quantitative variations depending on variety, maturity stage, geographical origin, and storage [30]. According to Thang et al. [31], the difference in total carbohydrate levels can be explained by fundamental changes in fruit parietal polysaccharides during maturation and storage of raw materials, in addition to extraction conditions.

### 2.7. Functional Properties

The analytical results in Table 7 show that the pectin extracted from clementine peels had a significantly higher gelling power (164 ° SAG) than the commercial one (150 ° SAG). This result is comparable to that of Benchabane [32], who reported that apple pectin has a gelling power of 177 to 220 ° SAG, while that of orange pectin varies between 170 and 200 ° SAG. This difference can be explained by the structural difference between apple and orange pectin (molecular weight, content of neutral oses, and presence or not of acetyl groups). Several studies have shown that the carbohydrate content, the distribution of non-esterified carboxyl groups, and the charge of pectin, together with the content of neutral oses and the presence of acetyl groups, have a strong influence on the structural and textural properties of pectic gels [33].

The emulsifying activity of the extracted pectin (38.46%) is significantly lower than that of the commercial pectin, which has a value of 51%. Our results are comparable with those obtained by Leroux et al. [34] and Yapo et al. [20], which are, respectively, 43.2 and 47.1% for beet pectin. According to Yapo et al. [20], this remarkable emulsifying activity of citric-acid-extracted pectin may be due to the fact that it is endowed with a tension-active activity that increases the viscosity of the aqueous phase and reduces the tendency for the emergence of dispersed oil globules. Also, it is mainly time and temperature that have a major influence on surface activity. The most surface-active pectins are those extracted at a temperature of 80 °C, at a pH of 1.5, and for one hour.

The Box–Behnken design was employed to investigate three the independent variables of temperature (60–100 °C), particle size (0.1–1 mm), and pH (1.5–3). The results demonstrated that increasing temperature and reducing both particle size and pH level significantly enhanced the extraction yield. The optimal conditions determined through response surface designs were pH = 1.5, T = 100 °C, and particle size = 0.1 mm, with a solid/liquid ratio of 1:50 (*m*/*v*) and an extraction time of 30 min. Under these conditions, the expected yield was 26.64%. In terms of functional properties, the extracted pectin exhibited a significantly higher gelling power (164 ° SAG) compared to commercial pectin (150 ° SAG). However, the emulsifying activity (38.46%) was lower than that of commercial pectin (51%).

## 3. Materials and Methods

### 3.1. Plant Material

Clementine fruits (*Citrus clementina*) of the Nova variety were harvested from the experimental station of the Regional Center of Agricultural Research of Tadla in Béni Mellal, Morocco (latitude: 32.26; longitude: −6.52). The fruit peels were manually removed from the pulp, washed, and blanched at 90 °C for 10 min to inactivate enzymes. Then, the peels were dried in a greenhouse at a controlled temperature (45 °C) for 8 days. The dried peels were ground with an electric grinder (Retsch SR300, Retsch GmbH, Haan, Germany) to obtain a fine and a homogeneous powder (0.1–1 mm). The moisture content of the obtained powder was 8.5%.

The powder was stored in hermetically sealed vacuum bags and protected from the light until experimental analysis.

### 3.2. Pectin Extraction

Hot acid extraction of the pectin was carried out according to Canteri-Schemin [19], with the following modifications. The dried and ground peel powder (1:50 *w*/*v*) was soaked in distilled water, and the pH was adjusted (pH 1.5–3) using an aqueous citric acid solution (25% *w*/*v*) and a pH meter (HANNA, hi 98161, Lingolsheim, France) under agitation. The solution was extracted at 60–100 °C for 30 min. The obtained pectic extract was kept at 4 °C for 24 h. Pectin was precipitated by mixing the extracted pectic juice with two volumes of 96% ethanol; the obtained pectic gel was washed with acetone (100%). Finally, the precipitated pectin was dried at 60 °C to a constant weight in a vacuum oven (Memmert, GmbH, VO29, Schwabach, Germany).

Commercial pectin (pastry pectin, Louis Francois-France) was analyzed for comparison.

### 3.3. Experimental Methodology

The pectin extraction parameters were optimized using RSM. A Box–Behnken design, an element of RSM, was used to identify the best experimental conditions.

Particle size (X1) (0.1–1 mm), pH (X2) (1.5–3), and extraction temperature (X3) (60–100 °C) were chosen as the independent variables. The extraction time was 30 min. For each factor, the experimental range was chosen based on the results from the literature and preliminary experiments.

In this study, to model the pectin extraction process, a linear model without interactions was used to approximate the relationship between the yield extraction and the three selected variables (X1, X2, and X3), as represented in Equation (2):

Y = b0 + b1*X1 + b2*X2 + b3*X3(2)

where Y is the calculated response function, and b1, b2, and b3 are the coefficients of the parameters X1, X2, and X3, respectively.

In order to validate the mathematical optimization model, influencing factors and variation ranges were chosen for this study, as illustrated in Table 8.

It is important to note that all computation and graphics in this study were performed using Design Expert Statistical Software 10 by Stat Ease, Inc. (Minneapolis, MN, USA).

### 3.4. Validation of the Model

Validation of the mathematical model is an important step. Thus, the validation was carried out by an appropriate analysis of variance (ANOVA), as described for the case of a composite design by Kamoun [35] and Masmoudi [2].

### 3.5. Chemical Characterization and Pectin Yield

#### 3.5.1. Moisture Content

To determine the moisture content (M%), one gram (1 g) of the sample was weighed (P1) in a tared porcelain crucible and then placed in an oven set at 105 °C. After 5 h, the sample was transferred to a desiccator for 30 min and weighed; the operation was repeated until obtaining a constant weight [36].

The moisture content was calculated as follows:

H(%) = (P2 − P1/P1) × 100(3)

where P1 is the weight of the sample before drying and P2 is the weight after drying in the oven.

#### 3.5.2. Ash Content

Ash content was determined using the AOAC method [37]. Pectin weighing 1 g was placed in a previously tared porcelain capsule and placed in a muffle oven (NAHITA serie 642, Gareoult, France) for 24 h at 550 °C. Then, the capsule was cooled in a desiccator and weighed. The ash content was calculated as the percentage of sample weights before and after muffle oven drying.

#### 3.5.3. Protein Content

The protein content was assessed using the Micro-Kjeldahl apparatus (Kel plus, Pelican, India), in accordance with the AOAC method [38]. A total of 0.2 g of pectin powder was digested in a solution of catalyst (1 g) and 5 mL of H_2_O_2_ and conc. H_2_SO_4_. The digested sample was allowed to boil before collecting the distillate of ammonia liberated in boric acid. The distillate was titrated with hydrochloric acid until the blue color was completely removed. The protein content was estimated using the following formula.

(4) $$N(\%)=\frac{\left(S-B\right)\times N\times 14.007\times Volume\;made\;(mL)}{Weight\;of\;sample\left(g\right)\times Volume\;taken\;(mL)}\times 100$$

where

*S* = mL of HCl required for sample titration;

*B* = mL of HCl required for blank titration;

*N* = normality of HCl (0.02 N).

Protein% = Nitrogen% × 6.25(5)

#### 3.5.4. Carbohydrate Content

The procedure described by Dubois in 1956 [39] was used to determine the amount of carbohydrate in the sample. A total of 0.5 mL of pectin extract and 0.5 mL of phenol solution at 5% was left for 15–30 min in darkness for ambient cooling; then, 3 ml of concentrated sulfuric acid was added. Galactose was used to generate the standard range, and a UV-Visible spectrophotometer was used to measure the optical density (OD) at 485 nm.

#### 3.5.5. Pectin Yield

The pectin extraction yield, the subject of this study, was calculated as follows:

Yield (%) = (weight of dried pectin/weight of dried clementine by-product) × 100%(6)

### 3.6. Functional Properties

#### 3.6.1. Determination of Gelling Power

The traditional SAG method was used to measure the strength of gels formed under the conditions of 65.0% soluble solids (sucrose), 0.70 wt% pectin, and a pH of 2.3 to determine the gelling capacity (or power) of pectin [40]. In order to prevent evaporation, the jelly mixture was placed entirely within a Ridgelimeter glass. It was then left undisturbed at ambient temperature for 2 h before aging for a further 22 h in a water bath at 30 °C. The gel was then delicately demolded onto a Ridgelimeter glass plate without causing any damage. The pointer of the device (Ridgelimeter) was gently lowered until it contacted the gel surface after exactly 2 min of standing, and the percentage of sagging under its specific gravity was measured. The following equation was used to determine the gelling power:

° SAG = (A/B) × F(7)

where A, B, and F are the amounts of sugar and pectin in gel and the factor of sagging percentage, respectively.

#### 3.6.2. Emulsifying Activity Analysis

The extracted pectin’s emulsion activity (EA) was determined using the method reported by Hosseini et al. [41]. In a brief, 5 mL of sunflower oil, 5 mL of extracted pectin solution (0.5% *w*/*v*), and 0.02% sodium azide (NaN_3_) were combined and subjected to 4000× *g* centrifugation for 5 min. Finally, EA was determined as follows:

EA(%) = VE/VT × 100%(8)

where VE is the volume of the emulsion layer, and VT is the total volume.

### 3.7. Statistical Analysis

All experiments were performed in triplicate, and the results were expressed as the mean ± standard deviation (SD). Analysis of variance was performed using (SPSS Corporation, Northampton, MA, USA). The significance level was set as *p* < 0.05 throughout the study.

## 4. Conclusions

The study demonstrates the feasibility of extracting pectin from *Citrus clementina* peel with interesting yield and functional properties. The best pectin yield (26.6%) was obtained using citric acid with extraction conditions optimized (pH 1.5, 100 °C, and particle size = 0.1 mm) using RSM, with a solid/liquid ratio of 1:50 (*m*/*v*) and an extraction time of 30 min. The extracted pectin exhibited a significantly higher gelling power (164 ° SAG) and interesting emulsifying activity (38.46%).

To further validate the practical application of the extracted pectin, future studies could focus on its application in food product preparation, such as for jams and jellies. It is essential to verify its effectiveness as a gelling and emulsifying agent and assess its environmental and chemical stability. By investigating these aspects, the potential benefits and applicability of *C. clementina* peel-extracted pectin can be better understood, paving the way for sustainable and functional food additives.

## Figures and Tables

**Table 1:** Experimental conditions of the Box–Behnken design and the corresponding experimental responses.

	Factor 1	Factor 2	Factor 3	Response 1
Std	X1: Particle Size	X2: pH	X3: Temperature	Yield
	mm		°C	%
1	0.1	1.5	80	25.4
2	1	1.5	80	18.7
3	0.1	3	80	10.5
4	1	3	80	3.8
5	0.1	2.25	60	17.4
6	1	2.25	60	10.8
7	0.1	2.25	100	18.7
8	1	2.25	100	19.3
9	0.55	1.5	60	21.5
10	0.55	3	60	6.5
11	0.55	1.5	100	22.8
12	0.55	3	100	7.8
13	0.55	2.25	80	14.7
14	0.55	2.25	80	14.6

Std: Sorting by standard.

**Table 2:** Fit summary.

Source	Sequential *p*-Value	Lack-of-Fit *p*-Value	Adjusted R^2^	Predicted R^2^	
Linear	<0.0001	0.0271	0.9130	0.8556	Suggested
2FI	0.3571	0.0271	0.9194	0.7681	
Quadratic	0.4610	0.0247	0.9212	0.6123	
Cubic	0.0247		0.9999		Aliased

2FI: Two-factor interaction.

**Table 3:** Lack-of-fit tests.

Source	Sum of Squares	df	Mean Square	F-Value	*p*-Value	
Linear	36.83	9	4.09	818.41	0.0271	Suggested
2FI	23.87	6	3.98	795.62	0.0271	
Quadratic	13.33	3	4.44	888.67	0.0247	
Cubic	0.0000	0				Aliased
Pure Error	0.0050	1	0.0050			

df: Degree of freedom.

**Table 4:** ANOVA for linear model.

Source	Sum of Squares	df	Mean Square	F-Value	*p*-Value	
Model	513.27	3	171.09	46.45	<0.0001	Significant
X1—Particle size	47.05	1	47.05	12.77	0.0051	
X2—pH	447.01	1	447.01	121.36	<0.0001	
X3—Temperature	19.22	1	19.22	5.22	0.0455	
Residual	36.83	10	3.68			
Lack of Fit	36.83	9	4.09	818.41	0.0271	Significant
Pure Error	0.0050	1	0.0050			
Cor Total	550.10	13				

**Table 5:** Fit statistics.

**Std. dev.**	**1.92**	**R^2^**	0.9330
**Mean**	15.18	**Adjusted R^2^**	0.9130
**C.V. %**	12.64	**Predicted R^2^**	0.8556
		**Adeq. Precision**	19.3009

Std. dev: Standard deviation. C.V. %: Coefficient of variation.

**Table 6:** Coefficients in terms of coded factors.

Factor	Coefficient Estimate	df	Standard Error	95% CI Low	95% CI High	VIF
Intercept	15.18	1	0.5129	14.04	16.32	
X1—Particle size	−2.43	1	0.6785	−3.94	−0.9131	1.0000
X2—pH	−7.48	1	0.6785	−8.99	−5.96	1.0000
X3—Temperature	1.55	1	0.6785	0.0381	3.06	1.0000

CI: Confidence interval. VIF: Variance inflation factor.

**Table 7:** Functional properties of extracted pectin compared to commercial pectin.

	Gelling Power	Emulsifying Activity
Extracted pectin	164° ± 2° SAG ^a^	38.46% ± 1.66% ^a^
Commercial pectin	150° ± 1° SAG ^a^	51% ± 2% ^b^

Values followed by the same letters (a,b) are not significantly different (*p* < 0.05). SAG: Strain-induced Alignment in a Gel.

**Table 8:** Variation factors of the most influential factors.

	Factors	Lower Level	Higher Level	Medium Level
X1	Particle size (mm)	0.1	1	0.55
X2	pH	1.5	3	2.25
X3	Temperature (°C)	60	100	80

## Data Availability

Data other than presented in this paper are available upon request. Please send all communications to Elfazazi Kaoutar at kaoutar.elfazazi@inra.ma or Azzouzi Hanane hananeazzouzi94@gmail.com.
